# Outcomes following nurse-led day-case paracentesis

**DOI:** 10.1016/j.clinme.2025.100538

**Published:** 2025-11-26

**Authors:** Mahir Yousuff, Pramudi Wijayasiri, Renee Ma, Rabiat Umar, Ripak Purbe, Nicola J. Monahan, Helen L. Garrity, Beverley J. Aram, Naaventhan Palaniyappan, Emilie Wilkes, Aloysious D. Aravinthan

**Affiliations:** a NIHR Nottingham Biomedical Research Centre, Nottingham University Hospitals NHS Trust and University, Nottingham, UK; b Nottingham Digestive Diseases Centre, School of Medicine, Translational Medical Sciences, University of Nottingham, Nottingham, UK

**Keywords:** Advanced chronic liver disease, Refractory ascites, Therapeutic paracentesis

## Abstract

**Background:**

Therapeutic paracentesis (TP) is a key symptom-relieving intervention in refractory ascites. In response to rising demand, Nottingham University Hospitals NHS Trust was among the first UK centres to introduce a nurse-led, day-case TP service. This study evaluates the complications and mortality associated with this service.

**Methods:**

Retrospective analysis was undertaken on patients with decompensated cirrhosis who underwent nurse-led day-case TP between 1 January 2017 and 31 December 2021. Clinical and outcome data were systematically collected.

**Results:**

A total of 2,530 TP procedures were performed on 340 patients. Minor (self-limiting) complications occurred in 54 cases (2.1%). In 22 cases (0.9%), patients were admitted to hospital within 7 days of TP – the reasons for admission were bacterial peritonitis (*n* = 11), abdominal pain (*n* = 3), and leakage of ascitic fluid (*n* = 7) or bleeding (*n* = 1) from the puncture site. There were no cases of abdominal visceral perforation or in-hospital deaths among those admitted.

About 290 patients were included in 1-year survival analysis. About 177 (61%) were alive 1 year after their first TP. The median time from first TP to death was 125 days (IQR 43–210). The 30- and 90-day mortality rates after TP were 21.0% and 33.1%, respectively. Older age (*p* < 0.001), hepatocellular carcinoma (*p* < 0.001) and ongoing alcohol use (*p* < 0.001) were independently associated with 1-year mortality.

**Conclusion:**

Nurse-led, day-case TP is an effective and safe approach for managing refractory ascites in decompensated cirrhosis. Mortality remains high, particularly in patients with hepatocellular carcinoma and ongoing alcohol use.

## Highlights

• About 10% of patients with cirrhosis and ascites require therapeutic paracentesis (TP).
• Nurse-led day-case services are being established due to rising demand for TP.
• Nurse-led services have similar morbidity risks to physician-led services.
• The cumulative yearly mortality rates of TP are demonstrated.

## Graphical abstract

**Figure:**
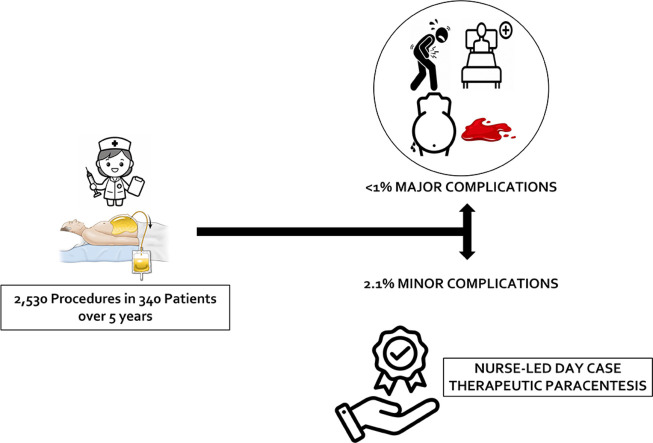

## Introduction

Chronic liver disease (CLD) is the second most common cause of premature death in the UK and the only major cause of death that is still increasing, with a 400% rise since 1970.^1^ CLD progresses through worsening liver injury, inflammation and fibrosis, leading to cirrhosis.^2^ Decompensation events are episodes of worsening liver function in individuals with cirrhosis, signalling a critical shift in disease trajectory. Ascites, hepatic encephalopathy, jaundice and variceal bleeding in a patient with cirrhosis are recognised as decompensation events. Among these, ascites – the accumulation of fluid in the peritoneal cavity – is the most common.^3^ Each year, 5–12% of patients with compensated cirrhosis progress to decompensation^4^, ^5^, ^6^; at least 50% of them develop ascites.^3^ The onset of decompensation significantly reduces the median survival from 12 to just 2 years.^6^

The development of ascites is driven by hepatic synthetic impairment and portal hypertension, reflecting the haemodynamic reorganisation that occurs in cirrhosis.^7^^,^^8^ This results in a 1- and 2-year mortality of 15% and 40%,^4^ respectively, which increases to >60% when complicated by hyponatraemia, hepatorenal syndrome, and/or superimposed spontaneous bacterial peritonitis (SBP).^9^

As per the National Institute for Health and Care Excellence (NICE) UK^10^ and European Association for the Study of the Liver (EASL)^11^ guidelines, graduated diuretic therapy and dietary sodium restriction are initial management strategies for ascites. As the disease progresses, renal sodium retention worsens, necessitating progressively higher doses of diuretics.^12^ Approximately 10% of patients with decompensated cirrhosis and ascites develop either intolerance or resistance to diuretics, resulting in refractory ascites.^12^ In addition to the complications associated with ascites, refractory ascites significantly reduces quality of life in various ways, including a persistent sense of fullness, anorexia, nutritional deficiencies, and the development of abdominal hernias.^13^ Regular therapeutic paracentesis (TP) is the mainstay of symptomatic management for patients with refractory ascites and, in a minority of cases, serves as a bridge to liver transplantation or transjugular intrahepatic portosystemic shunt (TIPS).^14^

Due to the increasing demand for TP services, a nurse-led, day-case paracentesis service was established at Nottingham University Hospitals NHS Trust (NUH) and other NHS trusts across the country over the past decade.^15^, ^16^, ^17^, ^18^, ^19^, ^20^ Studies have demonstrated a positive impact on patient waiting times,^15^^,^^21^ hospital bed pressures,^17^ length of hospital stay,^18^ hospital costs^17^^,^^20^ and patient satisfaction.^15^^,^^16^ However, morbidity and mortality outcomes associated with nurse-led, day-case paracentesis services for patients with advanced CLD have not been extensively studied. The aim of this study was to evaluate the outcomes of the nurse-led paracentesis service at NUH.

## Methods

### Patient selection and data collection

**Fig. 1:**
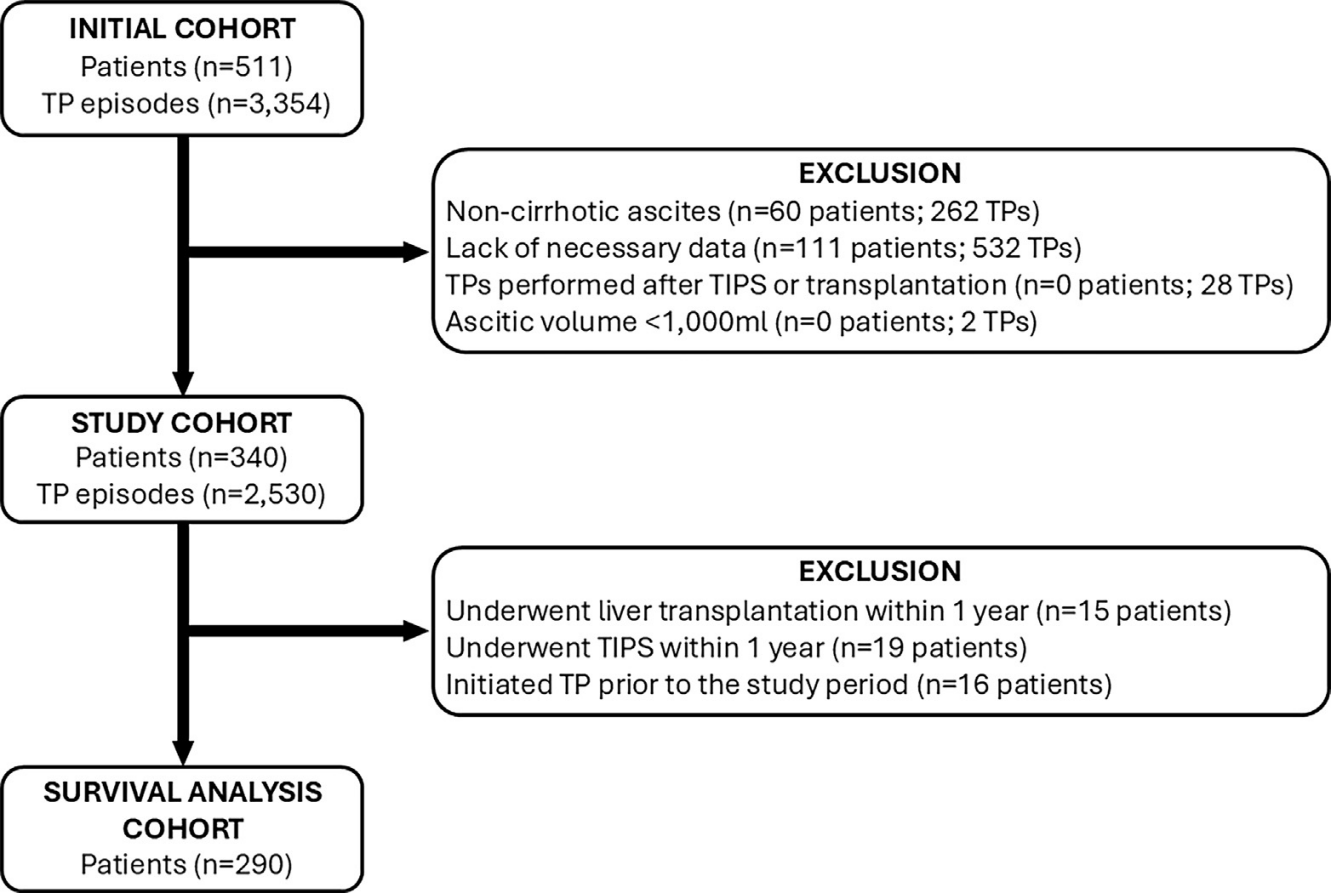
Flow diagram illustrating patient and therapeutic paracentesis (TP) episode selection for the study cohort. A total of 511 patients underwent 3,354 episodes of nurse-led day-case TP during the study period. After excluding patients with non-cirrhotic ascites, insufficient data, TP following liver transplantation or TIPS insertion, and TP episodes with an ascitic volume <1,000 mL, the final study cohort comprised 340 patients and 2,530 TP episodes.

Contemporaneously collected data from NUH, a high-volume tertiary hepatology unit in the UK, were retrospectively analysed. All adult patients (age ≥18 years) with decompensated CLD who underwent nurse-led, day-case TP during the 5-year study period (between 1 January 2017 and 31 December 2021) were included. Patients who underwent TP for non-cirrhotic causes of ascites (eg ascites related to malignancy or heart failure) and those who underwent diagnostic paracentesis were excluded (Fig. 1). Demographic and clinical data were extracted retrospectively from electronic medical records. This study was approved by the NUH Clinical Effectiveness Board (22-007C) as a service evaluation, waiving the requirement for individual informed patient consent to access medical records held within NUH.

### Nurse-led day-case TP

Historically, patients with decompensated CLD requiring TP were admitted as a day case, where the procedure was performed by a resident physician. In response to increasing demand and poor patient satisfaction, NUH established a nurse-led paracentesis service in September 2015. This service allows patients to contact the day-case unit directly when TP is needed or to schedule the procedure at regular intervals, such as fortnightly, if required. The service is staffed by two clinical nurse specialists (CNS) and one paracentesis assistant practitioner; both CNS were trained and assessed by consultant hepatologists to perform paracentesis independently. Training included a minimum of 30 supervised procedures, alongside instruction in liver anatomy and physiology. Should any issues or queries arise during a patient visit, the team has direct access to a designated hepatology consultant within the hospital, who can provide in-person support if necessary.

All TPs are performed under bedside ultrasound guidance (Sonosite®) using Biotech Neo Hydrophilic eight French drainage catheters (without a safety string). Drains are left *in situ* for a maximum of 4 hours or until the patient experiences symptomatic relief. For patients with impaired renal function, drainage is typically limited to 5 L. Before undergoing TP, recent blood test results – including haemoglobin, platelets, prothrombin time, urea, creatinine and estimated glomerular filtration rate – are reviewed to assess bleeding risk and the potential for post-paracentesis circulatory dysfunction (PPCD). Blood tests must have been taken within the past 3 months. Patients receive human albumin solution (HAS) for intravenous volume expansion in accordance with local protocol when more than 5 L is drained. The standard regimen is 100 mL of 20% HAS for every 2.5 L drained, to help prevent PPCD.^22^

### Definitions

The date of cirrhosis diagnosis was defined as the earliest recorded mention of confirmed cirrhosis in a radiology or histology report or in clinical correspondence.

The censoring date, defined as the last date on which survival outcomes were recorded, was 31 December 2022, ensuring a minimum follow-up period of 1 year from the study end date.

TP was defined as the removal of 1,000 mL or more of ascitic fluid in a single procedure to relieve ascites-related symptoms.

Any documented alcohol consumption, regardless of quantity, was considered indicative of ongoing alcohol use in patients with alcohol-related cirrhosis.

Any relevant complication that was contemporaneously recorded on electronic medical records was included. The following complications were considered associated with TP: post-TP bleeding at the puncture site, post-TP ascitic fluid leakage from the puncture site; perforation of abdominal viscera; and bacterial peritonitis diagnosed within 7 days of TP (but not identified in the ascitic fluid sample taken during the TP episode). A minor complication was defined as a self-limiting event requiring minimal medical intervention, such as puncture site pain, oozing or bleeding, or leakage of ascitic fluid that resolved spontaneously or with local treatment (eg glue therapy or application of an adhesive bandage). Major complications were those necessitating hospital admission and/or invasive interventions (eg angiography and embolisation) solely as a result of TP. Complications of CLD, such as electrolyte disturbances, renal impairment, hepatic encephalopathy and SBP, identified at the time of TP and requiring hospital admission, were not considered complications of the procedure.

### Outcomes

The study aimed to evaluate the safety and effectiveness of the service as defined by TP-related complication rates and long-term outcomes.

The primary outcome was the incidence of the aforementioned TP-related complications within 7 days of a TP episode.

Secondary outcomes included patient survival, as well as the incidence of liver transplantation, placement of a TIPS as a definitive treatment and the insertion of palliative long-term abdominal drains for ascites management. Patients who underwent liver transplantation, TIPS placement, or the insertion of long-term palliative abdominal drains within 1 month of starting TP were excluded from the survival analysis.

### Statistical analysis

Data are presented as the median and interquartile range (IQR) for continuous variables, or as numbers and percentages for categorical variables. Statistical analyses were carried out using GraphPad Prism 9 (San Diego, CA) for univariate analysis and descriptive statistics. The Mann–Whitney U test and chi-square test were used to compare data. Any variable with a *p*-value of <0.10 in the univariate analysis was included in a multivariable Cox proportional hazards model, employing a forward stepwise approach. All analyses were conducted using IBM SPSS Statistics for Windows, Version 29.0.2.0 (Armonk, NY). Statistical significance in univariate analysis was defined as a *p*-value <0.05. To identify factors independently associated with 1-year mortality, a multivariable binary logistic regression analysis was undertaken. Variables with a *p*-value <0.10 in univariate analysis were entered into the multivariable model. An association was considered independently significant only if the *p*-value reached the Bonferroni-adjusted threshold for statistical significance.

## Results

### Demographics and clinical characteristics

A total of 511 patients underwent 3,354 nurse-led day-case paracentesis procedures at NUH during the 5-year study period (Initial Cohort). Of these, 171 patients (comprising 824 procedures) were excluded due to non-hepatic causes of ascites, insufficient data, paracentesis following liver transplantation or TIPS, or having undergone diagnostic rather than therapeutic paracentesis (Fig. 1). Consequently, 340 patients, accounting for 2,530 TP episodes, met the eligibility criteria and comprised the study cohort. The demographic and clinical characteristics of this cohort are summarised in Table 1.

**Table 1:** Demographics and clinical characteristics of the entire study cohort (*n* = 340).

	All patients (*n* = 340) Median (IQR) or number (%)
Age at diagnosis (years)	58.3 (49.6–67.5)
Male sex	211 (62%)
Aetiology^a^	
ALD	251 (74%)
MASLD	90 (26%)
Viral hepatitis	15 (4.4%)
Autoimmune	17 (5%)
Other	10 (3%)
Presence of HCC	27 (8%)
Ongoing alcohol intake	121 (48.2%)
Diagnosis to first TP (days)	94 (5–813)
TP episodes per patient	3 (1–10)

	All therapeutic paracentesis (*n* = 2,530) Median (IQR) or number (%)
Volume drained (mL)	7,702 (5,919–9,715)
SBP within 7 days of TP	11 (0.4%)
TP-related admissions	22 (0.9%)

a The aetiology parameter includes both sole and concomitant aetiologies; as a result, the total number and percentages exceed the overall study cohort (*n* = 340) and 100%, respectively. Abbreviations: ALD, alcohol-related liver disease; HCC, hepatocellular carcinoma; MASLD, metabolic dysfunction-associated steatotic liver disease; TP, therapeutic paracentesis.

The median age at diagnosis of cirrhosis was 58.3 years (IQR 49.6–67.5). Nearly two-thirds of the patients were male (*n* = 211, 62.1%). Alcohol-related liver disease (ArLD) was documented as an aetiology in 251 (73.8%) of patients, either as a sole aetiology or in combination. Similarly, metabolic dysfunction-associated steatotic liver disease (MASLD) and chronic viral hepatitis were documented as aetiologies in 90 (26.5%) and 15 (4.4%) of patients, respectively. Hepatocellular carcinoma was diagnosed in 27 patients (7.9%) at some point during the study period.

The median number of TP episodes per patient was three (IQR 1–10), with 91 patients (26.8%) having only one TP episode, 171 patients (50.3%) having between two and 10 TP episodes, and 78 patients (22.9%) having 11 or more TP episodes during the study period. The median volume of ascites drained per episode was 7,702 mL (IQR 5,919–9,715). The median duration from diagnosis of cirrhosis to the initiation of TP was 94 days (IQR 5–813).

### Primary outcomes

Of the 2,530 TP episodes, 54 (2.1%) were complicated by minor ascitic fluid leak while four (0.16%) involved minor bleeding at the puncture site.

Twenty-two (0.9%) episodes resulted in hospital admission. The reasons for admission were ascitic fluid leakage from the puncture site (*n* = 7), bleeding from the puncture site (*n* = 1), post-procedure abdominal pain (*n* = 3), and bacterial peritonitis diagnosed within 7 days of TP (*n* = 11). The median length of hospital stay for these patients was 3 days (IQR 1.5–9.5). No in-hospital deaths occurred among these patients, and no cases of post-TP abdominal visceral perforation were recorded.

### Secondary outcomes

Of the 340 patients in the study cohort, only 290 were eligible for 1-year survival analysis. Fifty were excluded – 15 underwent liver transplantation, 19 had TIPS placement, and 16 had initiated TP prior to the study period.

**Fig. 2:**
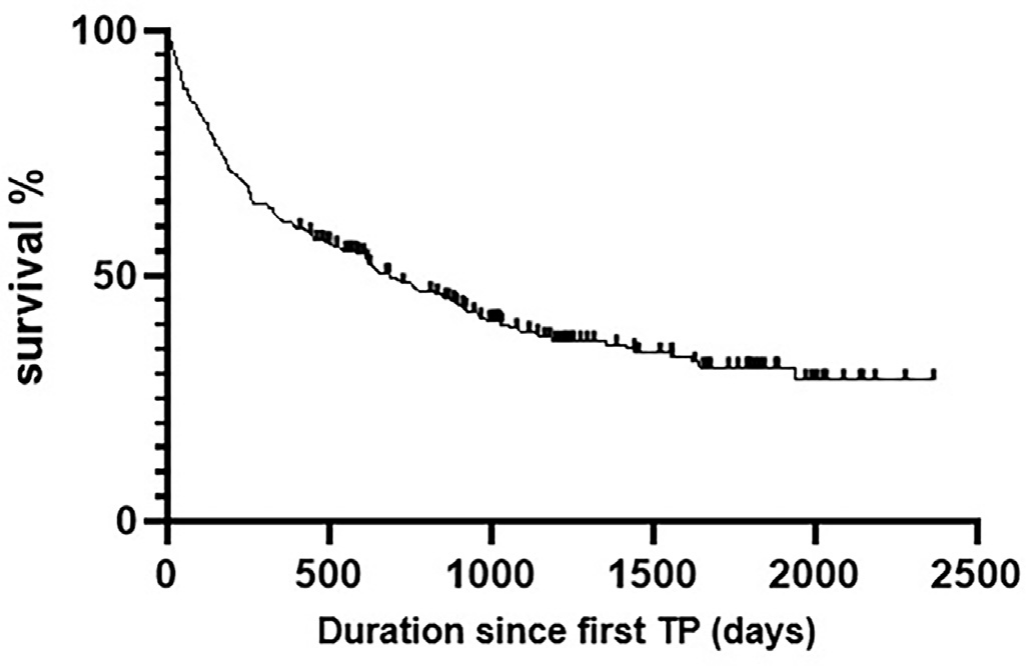
Overall survival from the time of first therapeutic paracentesis (TP) in the study cohort. Kaplan–Meier survival analysis showing cumulative survival of 290 patients undergoing TP, with follow-up extending more than 6 years.

Of the 290 patients included in the survival analysis, 177 (61%) survived for at least 1 year following their first TP (‘Survivors’). Among those who did not survive (‘Non-survivors’), the median time from first TP to death was 125 days (IQR 43–210). In this cohort, the 30- and 90-day mortality following a TP episode was 21% and 33.1%, respectively. Cumulative mortality after the first TP was 39% at 1 year, 51% at 2 years, and 57% at 3 years (Fig. 2).

On univariate analysis (Table 2), factors associated with 1-year mortality included older age at diagnosis (*p* = 0.0001), presence of hepatocellular carcinoma (HCC) (*p* = 0.0001), ongoing alcohol use within 1 year of the first TP in patients with alcohol-related liver disease (*p* = 0.01), a longer interval between cirrhosis diagnosis and first TP (*p* = 0.002), and fewer TP episodes per patient (*p* = 0.003).

**Table 2:** Demographics and clinical characteristics of patients included in the 1-year survival analysis (*n* = 290).

	All (*n* = 290) Median (IQR) or Number (%)	Survivors (*n* = 177) Median (IQR) or number (%)	Non-survivors (*n* = 113) Median (IQR) or number (%)	Univariate *p*-value	OR (95% CI)	*p*-value
Age at diagnosis (years)	59.0 (49.6–68.7)	56.8 (47.0–65.7)	64.3 (54.9–73.3)	**0.0001**^a^	1.060 (1.031–1.090)	**<0.001**
Male sex	179 (61.7%)	104 (58.8%)	75 (66.3%)	0.19		
Primary aetiology				0.051^a^	1.641 (0.776–3.467)	0.201
ALD	188 (64.8%)	125 (70.6%)	63 (55.8%)			
MASLD	73 (25.2%)	34 (19.2%)	39 (34.5%)			
Viral hepatitis	13 (4.5%)	9 (5.1%)	4 (3.5%)			
Autoimmune	13 (4.5%)	7 (4.0%)	6 (5.3%)			
Other	3 (1.0%)	2 (1.1%)	1 (0.9%)			
Presence of HCC	24 (8.3%)	2 (1.1%)	22 (19%)	**0.0001**^a^	20.333 (4.194–98.574)	**<0.001**
Ongoing alcohol intake	86 (29.7%)	43 (24.3%)	43 (38.1%)	**0.01**^a^	4.857 (2.458–9.599)	**<0.001**
Diagnosis to first TP (days)	74 (5–662.5)	35 (0–409)	289 (30.5–1,118)	**0.002**^a^	1.000 (1.000–1.001)	0.011

a All parameters with a *p*-value <0.01 in the univariate analysis were included in the multivariate model. Statistical significance in the univariate analysis was defined as a *p*-value <0.05. In the multivariate analysis, significance was determined using a Bonferroni-adjusted threshold (*p* < 0.01). Abbreviations: ALD, alcohol-related liver disease; HCC, hepatocellular carcinoma; MASLD, metabolic dysfunction-associated steatotic liver disease; TP, therapeutic paracentesis.

On multivariate analysis (Table 2), independent factors associated with 1-year mortality, following Bonferroni correction for statistical significance, included older age (OR 1.060, 95% CI 1.031–1.090, *p* < 0.001), presence of HCC (OR 20.333, 95% CI 4.194–98.574, *p* < 0.001) and ongoing alcohol use (OR 4.857, 95% CI 2.458–9.599, *p* < 0.001).

Of the entire study cohort (*n* = 340), 20 patients (5.9%) underwent liver transplantation during the study period, following a median of eight TP procedures (IQR 3–14). A further 31 patients (9.1%) underwent TIPS placement after a median of nine procedures (IQR 3–12). In addition, seven patients (2.1%) received palliative long-term abdominal drains after a median of 17 TP procedures (IQR 7–31).

## Discussion

This study examines the characteristics and clinical outcomes of patients with decompensated cirrhosis who underwent nurse-led, day-case TP. It is among the first to assess morbidity and mortality outcomes associated with such a service. Notably, complication rates were comparable to those reported in physician-led services, highlighting the safety and viability of nurse-led models. Independent predictors of 1-year mortality – older age, presence of hepatocellular carcinoma (HCC), and ongoing alcohol consumption – were consistent with well-established risk factors in this high-risk population.

In an earlier study conducted prior to the introduction of nurse-led TP services, minor complications were reported in 8.9% of procedures.^23^ This study demonstrated a lower incidence of minor complications, with only 2.1% of TP episodes followed by self-limiting bleeding or leakage of ascitic fluid. The risk of bacterial peritonitis following TP, as well as bleeding requiring hospital admission, was comparable to rates reported in similar patient cohorts.^24^, ^25^, ^26^ Organ perforation, a recognised but rare complication, was estimated to occur in fewer than 1% of cases as early as 1978,^27^ before the introduction of safer TP drainage catheters^28^; as expected, no cases of organ perforation were observed in our cohort. Previous studies have shown no significant difference in complication rates between TPs performed by physicians and those performed by trained nurses,^19^^,^^29^ and the findings of this study further support this evidence, reinforcing the safety of nurse-led TP services.

The median time from the first TP episode to death was 125 days, corresponding to a 1-year mortality of 39%. The 30-day and 90-day mortality following TP was 21% and 33.1%, respectively. These findings align with previously reported 6–12-month mortality of up to 50% in patients with refractory ascites.^30^, ^31^, ^32^ To our knowledge, medium-term mortality risk associated with TP in refractory ascites has not been previously described. As such, the findings from this study may serve as a useful benchmark. Furthermore, as the survival analysis excluded patients who may have undergone TP via physician-led services prior to the study period, the outcomes presented here provide an accurate representation of the nurse-led service.

Older age, ongoing alcohol consumption, and the presence of HCC were independently associated with increased mortality in this study. Age has previously been identified as an independent risk factor for mortality in CLD, likely due to an exaggerated inflammatory response to liver injury,^33^ diminished hepatic blood flow^34^ and an increased burden of senescent cells.^35^ Sustained alcohol abstinence has been shown to improve prognosis at all stages of cirrhosis.^36^ HCC, which develops in 1–3% of patients with cirrhosis annually^37^, ^38^, ^39^ and carries a 5-year cumulative incidence of 5–30%,^40^ is associated with particularly poor outcomes, especially in those with decompensated disease.^41^^,^^42^ The findings of this study are consistent with the existing literature and further reinforce the prognostic significance of these well-established risk factors.

There are necessary biases when it comes to selecting patients suitable for nurse-led day-case TP and the study findings will reflect this select cohort, namely, patients would have had to be ambulatory and cognisant enough to attend the unit themselves at the appropriate time. The retrospective nature of this study presents several limitations. Obtaining accurate information on drinking habits and the exact date of cirrhosis diagnosis was challenging, particularly if the diagnosis occurred outside NUH or before the introduction of electronic hospital records in 2011, which may have influenced the results. Furthermore, cirrhosis is often diagnosed incidentally when patients present with its complications, making the date of diagnosis somewhat arbitrary. While it was possible to comment on the proximity of death to a paracentesis episode, the cause of death could not be determined. Complications were incompletely recorded in the medical notes and had to be cross-referenced with departmentally held nursing records, potentially leading to some complications being missed. Post-procedure pain was not routinely recorded unless it resulted in hospital admission, which may have led to an underestimation of the minor complication rate. Cost effectiveness is difficult to assess within the UK NHS setting in which these data were collected – this is another limitation of this study.

Nevertheless, this study underscores the success of a nurse-led day-case TP service in terms of clinical outcomes, contributing to the growing body of evidence that such services improve hospitalisation rates and patient satisfaction. The study was conducted within a high-volume service, with over 2,500 TP episodes performed over 5 years. The findings should offer reassurance to other centres considering the development of similar services and may provide grounds to upscale existing services, for example, by offering weekend or community-based units. Based on these results, patients referred to a comparable day-case paracentesis service can be counselled on the mortality risk and 30- and 90-day morbidity risk associated with undergoing nurse-led paracentesis.

## CRediT authorship contribution statement

**Mahir Yousuff:** Writing – original draft, Software, Project administration, Formal analysis. **Pramudi Wijayasiri:** Writing – original draft, Project administration, Methodology, Data curation. **Renee Ma:** Writing – review & editing, Investigation. **Rabiat Umar:** Writing – review & editing, Investigation. **Ripak Purbe:** Writing – review & editing, Investigation. **Nicola J. Monahan:** Writing – review & editing, Data curation. **Helen L. Garrity:** Writing – review & editing, Resources, Data curation. **Beverley J. Aram:** Writing – review & editing, Resources, Data curation. **Naaventhan Palaniyappan:** Writing – review & editing, Visualization, Conceptualization. **Emilie Wilkes:** Writing – review & editing, Methodology, Conceptualization. **Aloysious D. Aravinthan:** Writing – review & editing, Supervision, Methodology, Formal analysis, Conceptualization.

## Declaration of competing interest

The authors declare that they have no known competing financial interests or personal relationships that could have appeared to influence the work reported in this paper.
